# Toward Predicting Solubility of Arbitrary Solutes in Arbitrary Solvents: Prediction of Density and Refractive Index Using Machine Learning Algorithms with Global Sensitivity Analysis

**DOI:** 10.1021/acs.oprd.6c00166

**Published:** 2026-06-12

**Authors:** Brian Hu, Jingchen Zhai, Xiguang Qi, Xibing He, Nick X. Wang, Junmei Wang

**Affiliations:** † Department of Pharmaceutical Sciences and Computational Chemical Genomics Screening Center, School of Pharmacy, 6614 University of Pittsburgh, Pittsburgh, Pennsylvania 15261, United States; ‡ Small Molecule Discovery, Eli Lilly and Company, Lilly Corporate Center, Indianapolis, Indiana 46285, United States

**Keywords:** density, refractive index, machine learning, global sensitivity analysis, data curation

## Abstract

Density and refractive index (nD) are routinely used in organic process development to support solvent selection, mixture formulation, phase behavior analysis, and rapid assessment of composition during scale-up and manufacturing. Reliable knowledge of these properties enables informed process decisions when chromatographic or spectroscopic methods are impractical or unavailable, particularly at the early development stages. In addition, the two properties are strongly related to the van der Waals (VDW) energy of a molecule. Thus, accurate prediction of these two properties has great value in both organic process development, molecular mechanics force field development, and solvation free energy and solubility prediction of any arbitrary molecules. In this study, we gathered molecular characteristic information on roughly 5000 organic compounds for density records and 4000 organic compounds for nD values. Subsequently, the distinct GAFF (General AMBER Force Field) descriptors and RDKit descriptors of the compounds were generated and then applied to train various prediction models with a variety of machine learning (ML) algorithms for both properties, respectively. As a result, both GAFF and RDKit descriptors yielded various robust models with low average percent errors (APE), low root-mean-square errors (RMSE), and high correlation coefficients R^2^, while RDKit showed slightly better performance for predicting both properties. For each property, we further optimized the best-performing model and conducted both global sensitivity analysis (GSA) and Shapley analysis to identify specific features and feature groups that outstandingly contributed to model robustness. We next investigated the effect of temperature on model construction for each property by developing two additional models using a subset of data with available temperature information: one incorporating temperature as a feature and the other excluding it. Finally, we identified 72 outliers for the density property and performed molecular dynamics (MD) simulations of the pure liquids to predict their densities. We also conducted a thorough literature search for alternative experimental measurements. For most of these outliers, alternative values were found, and the updated experimental densities showed much better agreement with both the MD-predicted values and the AI model predictions. For density, the best-performing AI model has an average percent error (APE) of 3.15% for the test set if including the outliers and 2.67% if not. For nD, the best-performing model achieves an APE of 0.53% for the test set. The successful prediction of the two key molecular properties paves the road toward accurately predicting the solubility of an arbitrary solute in an arbitrary solvent, an endeavor that not only facilitates the pharmaceutical industry to develop better drug candidates but also increases efficiency regarding overall wet lab work. Certainly, robust ML models can be applied in organic process development.

## Graphical abstract

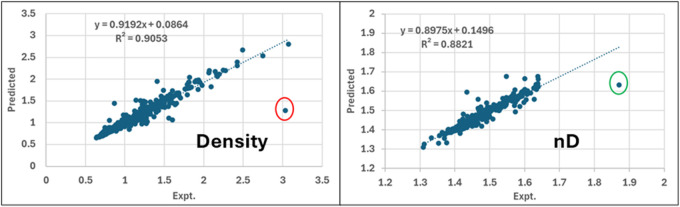

## 1 Introduction

Density and refractive index (nD) are process-relevant physical properties widely used in organic processes and development to support solvent selection, mixture formulation, phase behavior analysis, and composition assessment during scale-up and manufacturing. However, experimental measurement of these properties for novel intermediates, solvent systems, and early-stage compounds is often time-consuming and impractical during process development. Accurate, generalizable *in silico* prediction of density and refractive index can therefore provide valuable prior knowledge to guide the process design and decision-making.

The accurate prediction of two properties also plays an essential role in molecular design, which enables the prediction of the solvation free energy of a molecule using an implicit model. Solvation free energy is an essential descriptor for the accurate prediction of an arbitrary molecule’s solubility in a given solvent.^1^ The calculation of solvation free energy can be split into two parts, which are the nonpolar (np) and polar (p) (eq 1) terms.^2^

1 $$\Delta G=\Delta G_{\mathrm{np}}+\Delta G_{p}$$

The nonpolar term can be further decomposed into cavity (cav) and van der Waals dispersion (vdW) terms and is usually estimated using solvation-accessible surface area (SASA) (eq 2) with a linear relationship.^2–4^ The surface tension parameter, γ in eq 2, is a solvent-dependent property determined by factors including molecular polarizability, number of rotatable bonds, conformational entropy, heat of vaporization, and refractive index.

2 $$\Delta G_{\mathrm{np}}=\gamma \mathrm{SASA}+b$$

On the counterpart, the calculation of polar solvation energy is commonly estimated with the Poisson-Boltzmann (PB) theory or a generalized-Born (GB) model.^4–7^ To point out, the PB/GB calculation is significantly affected by the dielectric constant of the solute.^8^ The compound polarizability, which can be derived from the Lorentz–Lorenz eq (eq 3),^9^ can facilitate the determination of dielectric constant and surface tension parameter.^9,10^ Importantly, the density of compound and the index of refraction are key factors to calculate compound electronic polarizability (eq 3),^11^

3 $$\alpha =\frac{3}{4\pi }\frac{M}{\rho_{M}}\frac{n^{2}-1}{n^{2}+2}$$

where α represents electronic polarizability, *M* stands for molar mass, ρ is the density of the compound, and *n* is the index of refraction (nD).

Consequently, obtaining compound density and refractive index is vital to facilitate the estimation of solvation free energy for given compounds, which is both important and difficult to predict (Figure S1). Moreover, density and refractive index are vital properties for compounds to determine the molecular properties.

To date, the public domain includes only a few research studies of the compound density prediction. One main method to get first estimation of the density of compounds is structure-based calculation (*R* ^2^ = 0.982) on 166 compounds,^12^ which concludes atoms in different weight group and needs further calculation correction according to compound function groups. Another semiempirical method can calculate rough density for organic compounds according to atom elemental ratios,^13^ with the limitation of only being applicable to restricted particle components. Barley et al.^14^ compared composition-based methods for estimating liquid densities of organic compounds relevant to atmospheric aerosols and identified the most accurate approach. These methods rely on chemical structure or functional group composition to predict density. They also found that hygroscopic growth is relatively insensitive to the density, suggesting that a single approximate value can be used without introducing significant errors. The machine learning (ML) method has also been preliminarily applied to density prediction,^15^ and further model performance is summarized in Table 1.

**Table 1:** Current Studies from the Public Domain regarding the Prediction for Density and Refractive Index (nD)

property	method	model performance^a^	reference	additional performance/comments	data set size	Year
density	structure-based	*R* ^2^ = 0.982	^12^		166	1994
density	semiempirical	calculation accuracy = 12%	^13^	28 out of 31 compounds with CI within 12%	31	2012
density	machine learning	*R* ^2^ = 0.997, RMSE = 9.2 kg/m^3^	^15^		730 compounds with 5634 values	2012
nD	QSPR model	*R* ^2^ = 0.955	^20^		183 polymers	2002
nD	QSPR model	*R* ^2^ = 0.940	^17^		95 amorphous polymers at room temperature (298 K)	1998
		average prediction error = 0.9%				
nD	QSPR model	*R* ^2^ = 0.91	^21^	prediction of refractive index at 589 nm	126 compounds	2008
nD	fragment group contribution model	*R* ^2^ = 0.898	^18^		11,918 organic compounds	2014
		RMSE = 0.02				
nD	machine learning	mean percent error = 5.60%	^19^		6721 compounds with 49,076 records	2022
		mean absolute error = 0.151 for the best model				

a If the prediction is conducted with a machine learning method, the model performance is referred to the prediction of the test set result.

Current knowledge of the calculation for refractive index is also limited if the compound density is unknown.^16^ A couple of studies for nD estimation, which relies on compound structure information, include quantitative structure–property relationship (QSPR) model prediction,^17^ organic compound fragment group contribution model prediction,^18^ as well as recently developed ML algorithm prediction.^19^ Detailed model performance and data set size details are also summarized in Table 1.

Machine learning (ML), as a rapidly developing computational tool to facilitate chemical research, has great potential to reduce the cost and accelerate the process of research.^22,23^ The sophisticated algorithms of ML methods enable computers to process existing data and discover underlying data patterns.^24^ This technique has been applied to conduct prediction in many research fields, including chemistry, biology, and medical science.^25–27^ In this study, we applied ML functions for the prediction of compound density and refractive index (nD).

The objective of this project is to develop ML-based models to accurately predict the density and refractive index of compounds, thereby laying the foundation for solvation free energy calculations using physics-based methods and ultimately enabling the prediction of solubility for arbitrary solutes in arbitrary solvents. This work also represents a first step toward predicting the density and refractive index of mixtures, which are critical for automating process design.

## 2 Methods

### 2.1 Data Preparation

Our data library was derived from CRC Handbook of Chemistry and Physics [92nd edition, 2011–2012], which contains the information on 10,583 compounds. From this data source, 4896 compounds with valid density records measured under 1 atm and 4091 compounds with nD measurements were collected to conduct further study. Compounds were divided into training and external test sets at approximately an 85:15 ratio, with test compounds randomly sampled. For density model generation, we also summarized those compounds from the *CRC Handbook* with density values measured under higher pressure (>1 atm). A summary of data allocation for the density model is listed in Table 2. It is emphasized that Sets B–D in Table 2 were used as external test sets for model evaluation and were not involved in model training or construction. The Chemical Abstracts Service Registry Numbers (CAS RN) for all the compounds were collected and utilized to download, check, or generate compound structures in SMILES string or Sybyl Mol2 format from various online sources and further transfer them into descriptors.

**Table 2:** Data Summary for Density and nD. Data Sets A, B, C, and D Are for Density Models and Sets E and F Are for nD Models. All Data Come from CRC Handbook

data set	properties	purpose	data size
Set A	density	training set	4000
Set B	density	test set 1	678
Set C	density	test set 2	188
Set D	density	test set (high pressure)	30
Set E	nD	training set	3691
Set F	nD	test set	400

### 2.2 Model Building, Training, and Analyzing

Two descriptor systems, including the general AMBER force field (GAFF) descriptors and RDKit descriptors,^28^ were applied either independently or simultaneously to investigate model performance. The GAFF descriptors were generated with the Antechamber module of AMBER 18.^29,30^ The RDKit descriptors were generated by utilizing the Python package of RDKit.^31^ It is noted that descriptor generation using GAFF and RDKit was unsuccessful for a subset of compounds, resulting in different numbers of entries in the training and test sets for the two descriptor types (Table 3). Data standardization and dimensional reduction were performed prior to the model construction.

**Table 3:** The Performance of Machine Learning Models for Density Prediction on Set A and Set B^a^ ^,^ ^b^

data set	including outliers	model	no. data	ASE	APSE	AUE	RMSE	APE	*R* ^2^
Set A	No	GAFF_M1	3941	–0.002	–0.244	0.020	0.030	1.801	0.9910
Set A	No	GAFF_M2	3890	0.000	–0.046	0.012	0.019	1.136	0.9964
Set A	No	GAFF_M3	3890	0.000	–0.063	0.014	0.023	1.354	0.9947
Set A	No	RDKIT_M1	3940	0.000	–0.022	0.002	0.005	0.176	0.9998
Set A	No	RDKIT_M2	3889	0.000	–0.002	0.000	0.002	0.037	1.0000
Set A	No	RDKIT_M3	3889	0.000	–0.008	0.002	0.004	0.144	0.9998
Set B	No	GAFF_M1	678	0.002	–0.180	0.041	0.100	3.371	0.9053
Set B	No	GAFF_M2	671	0.006	0.156	0.037	0.100	3.070	0.9059
Set B	No	GAFF_M3	671	0.005	0.074	0.039	0.100	3.206	0.9062
Set B	No	RDKIT_M1	678	0.001	–0.321	0.037	0.097	2.969	0.9101
Set B	No	RDKIT_M2	671	0.004	–0.081	0.034	0.098	2.674	0.9107
Set B	No	RDKIT_M3	671	0.003	–0.188	0.036	0.097	2.860	0.9109
Set A	Yes	GAFF_M1	4000	0.000	–0.177	0.023	0.047	2.034	0.9795
Set A	Yes	GAFF_M2	3949	0.003	0.062	0.016	0.048	1.413	0.9782
Set A	Yes	GAFF_M3	3949	0.003	0.049	0.019	0.049	1.628	0.9777
Set A	Yes	RDKIT_M1	3999	0.000	–0.016	0.002	0.006	0.189	0.9997
Set A	Yes	RDKIT_M2	3948	0.002	0.092	0.005	0.045	0.333	0.9811
Set A	Yes	RDKIT_M3	3948	0.003	0.095	0.006	0.045	0.441	0.9811
Set B	Yes	GAFF_M1	691	0.002	–0.303	0.048	0.113	3.868	0.8796
Set B	Yes	GAFF_M2	684	0.006	0.101	0.043	0.114	3.537	0.8800
Set B	Yes	GAFF_M3	684	0.004	–0.049	0.046	0.113	3.701	0.8806
Set B	Yes	RDKIT_M1	691	0.001	–0.411	0.043	0.109	3.420	0.8879
Set B	Yes	RDKIT_M2	684	0.004	–0.151	0.040	0.110	3.148	0.8863
Set B	Yes	RDKIT_M3	684	0.003	–0.274	0.042	0.109	3.315	0.8882

a Definitions of performance metrics, ASE, APSE, AUE, RMSE, APE, and *R* ^2^ are provided in the footnote. The reported dataset sizes here correspond to the subset of molecules retained after descriptor generation; compounds for which descriptors could not be computed successfully were excluded from subsequent modeling.

b ASE, Average difference; APSE, average percent difference; AUE, average unsigned error; RMSE, root-mean-square error; APE, average percent error; *R* ^2^, correlation coefficient square.

MATLAB (R2024a) was used in this study, and the regression learner module was employed for model development. The machine learning algorithms evaluated are given in Tables S1 and S6. Model construction for each training set consisted of two main steps. First, the optimal algorithm was selected from 28 candidates based on the lowest root-mean-square error (RMSE). Specifically, the training data were randomly partitioned into 10 disjoint subsets for 10-fold cross-validation. For each fold, a model was trained on nine subsets and evaluated on the remaining subset, and the final validation error was computed as the average RMSE across all of the folds. The algorithm with the lowest average validation RMSE was selected as the best-performing method.

Second, using this selected algorithm, we retrained 20 independent models. The final model was chosen as the one with the lowest RMSE among these 20 runs. In each retraining step, the 10-fold partitions were regenerated randomly, leading to different data splits and potentially different optimized hyperparameters. Consequently, the resulting model performance varied across runs even for the same algorithm.

The model performance was evaluated by the root-mean squared error (RMSE), average percent error (APE), and correlation coefficient (R-square value) between the experimentally obtained property values and the prediction values. The RMSE and APE were calculated using the equations below, and R-square values were reported utilizing the embedded calculation function in MATLAB.

4 $$\mathrm{RMSE}=\sqrt{\frac{\sum_{i=1}^{N}{\left({\mathrm{predicted}}_{i}-{\mathrm{experimental}}_{i}\right)}^{2}}{N}}$$

5 $$\mathrm{APE}=\frac{1}{N}\sum_{i=1}^{N}\left|\frac{{\mathrm{predicted}}_{i}-{\mathrm{experimental}}_{i}}{{\mathrm{experimental}}_{i}}\right|\times 100\%$$

### 2.3 Feature Analysis of Machine Learning Models

We conducted feature analyses to evaluate the impact of descriptors on model performance for the GAFF_M1 and RDKIT_M1 models for both the density and refractive index (nD). Two complementary approaches were applied: the Shapley analysis and global sensitivity analysis (GSA). Shapley analysis is widely used to interpret the major contributions of ML models.^32^ However, it is computationally demanding, and its interpretability can be limited when descriptors are highly correlated, as is common with RDKit descriptors and molecular fingerprints. In contrast, GSA estimates the impact of descriptor groups on model performance and is better suited to addressing issues of computational cost and descriptor correlation.^33^

In this study, Shapley analysis was performed using built-in MATLAB functions, while GSA was conducted using in-house scripts implemented in MATLAB through a multistep procedure. First, correlated descriptor groups were identified through correlation analysis using a squared correlation coefficient (*R* ^2^) threshold of 0.5. A descriptor was assigned to a group if it had an *R* ^2^ ≥ 0.5 with at least one other descriptor in that group, though not necessarily with all descriptors in the same group. Each descriptor group was then excluded in turn during repeated (10-run) model reconstruction using the same ML algorithm to evaluate its impact on model performance. The mean RMSE and *R* ^2^ values from these runs were calculated and compared with those of the baseline model, in which all descriptors were retained.

### 2.4 Molecular Dynamics Simulations

For the density, we also identified several outliers in both the GAFF_M1 and RDKIT_M1 models. Molecular dynamics (MD) simulations were performed to compute the pure-liquid densities of these outlier compounds using an established protocol.^34,35^ Briefly, a simulation box containing hundreds to thousands of molecules of the target compound was generated using AmberTools.^36^ The system was first energy-minimized for 20 000 cycles, followed by a 100 ps MD run to relax the structure. A subsequent 2.5 ns MD simulation was carried out to ensure equilibration of the liquid phase, followed by an additional 2.5 ns production run for the density calculation. The MD system was described by the GAFF2 force field^29^ with the ABCG2 partial charge model,^34,38^ and residue topology and additional force field parameter files were generated using the Antechamber module^39^ in AmberTools.^36^

All MD simulations were conducted at the corresponding experimental temperature. A time step of 1.0 fs was used during the relaxation stage and 2.0 fs during the equilibration and production stages. Simulations were performed in the NPT ensemble using isotropic pressure scaling. Short-range interactions were computed with a 10 Å cutoff, and long-range electrostatic interactions were treated using the particle mesh Ewald (PME) method with default settings.^40^

## 3 Results

For GAFF descriptors, which share a consistent definition (i.e., each descriptor represents the count of atoms assigned to a given atom type), they were used without further transformation. For RDKIT descriptors, we evaluated the effect of normalization on model performance and found only minor differences for both density and nD; therefore, the original values were retained for model training. We also investigated dimensionality reduction using principal component analysis (PCA) but observed no significant improvement in the performance. Accordingly, the final models were trained without the application of dimensionality reduction.

### 3.1 Density Prediction

The density distribution of all the compounds involved in this study is shown in Figure S2. For the data from *CRC Handbook of Chemistry and Physics* [92nd edition, 2011–2012], the distributions of compound density have a similar pattern between the training set (Set A) and test set (Set B), indicating that the test set (Set B) can exactly reflect model robustness generated from the training set (Set A).

We first applied all regression models to set A using both GAFF and RDKit descriptors to identify the most promising ML algorithm as the candidate model type. In total, 28 ML algorithms across eight families were evaluated: linear regression (4), tree-based models (3), support vector machines (SVM, 6), efficient linear models (2), ensemble methods (2), Gaussian process regression (GPR, 4), neural networks (NN, 5), and kernel methods (2). For both descriptor types, GPR achieved the best validation performance, with rational quadratic GPR performing best for GAFF and exponential GPR for RDKit. As shown in Table S1, the top-performing algorithm families include GPR, NN, and ensemble models for both GAFF and RDKit, while SVM also delivers a satisfactory performance when trained with RDKit descriptors.

After identifying the best-performing model, we conducted 20 independent training runs with different random seeds to further optimize the model for the same machine learning algorithm. The best model obtained after this reoptimization is designated as the M1 model, including GAFF_M1 and RDKIT_M1 for the GAFF and RDKit descriptors, respectively. Multiple performance metrics were used to evaluate model performance, including average signed error (ASE), average percent signed error (APSE), average unsigned error (AUE), root-mean-square error (RMSE), average percent error (APE), and the coefficient of determination (*R* ^2^), even though the models were trained to minimize RMSE. To demonstrate the robustness of our model training process for density, we illustrated the distributions of the RMSE values of 20 independent training runs for M1 models and M2 and M3-series models introduced later (Figure S3). The corresponding mean and standard deviation values are displayed at the top of each chart.

Although RDKIT_M1 shows slightly better predictive accuracy than GAFF_M1, the latter demonstrates greater robustness, as reflected by smaller differences in RMSE and APE between the training and test sets. Detailed data, including each compound’s CAS ID, experimental value and measurement temperature, SMILES string, and predicted densities from all ML models, are provided in Tables S2–S5 for Sets A–D. Overall model performance is summarized in Tables 3 and 4. Scatter plots of predicted versus experimental values for the test sets using GAFF_M1 and RDKIT_M1 are shown in Figure 1. Notably, the outliers in Figure 1 correspond to the same molecule, tetrafluoromethane, which is a gas at 25 °C. The CRC Handbook lists its density as 3.034 g/cm^3^; the correct value, reflecting its gaseous state, should be 0.003034 g/cm^3^.

**Figure 1:**
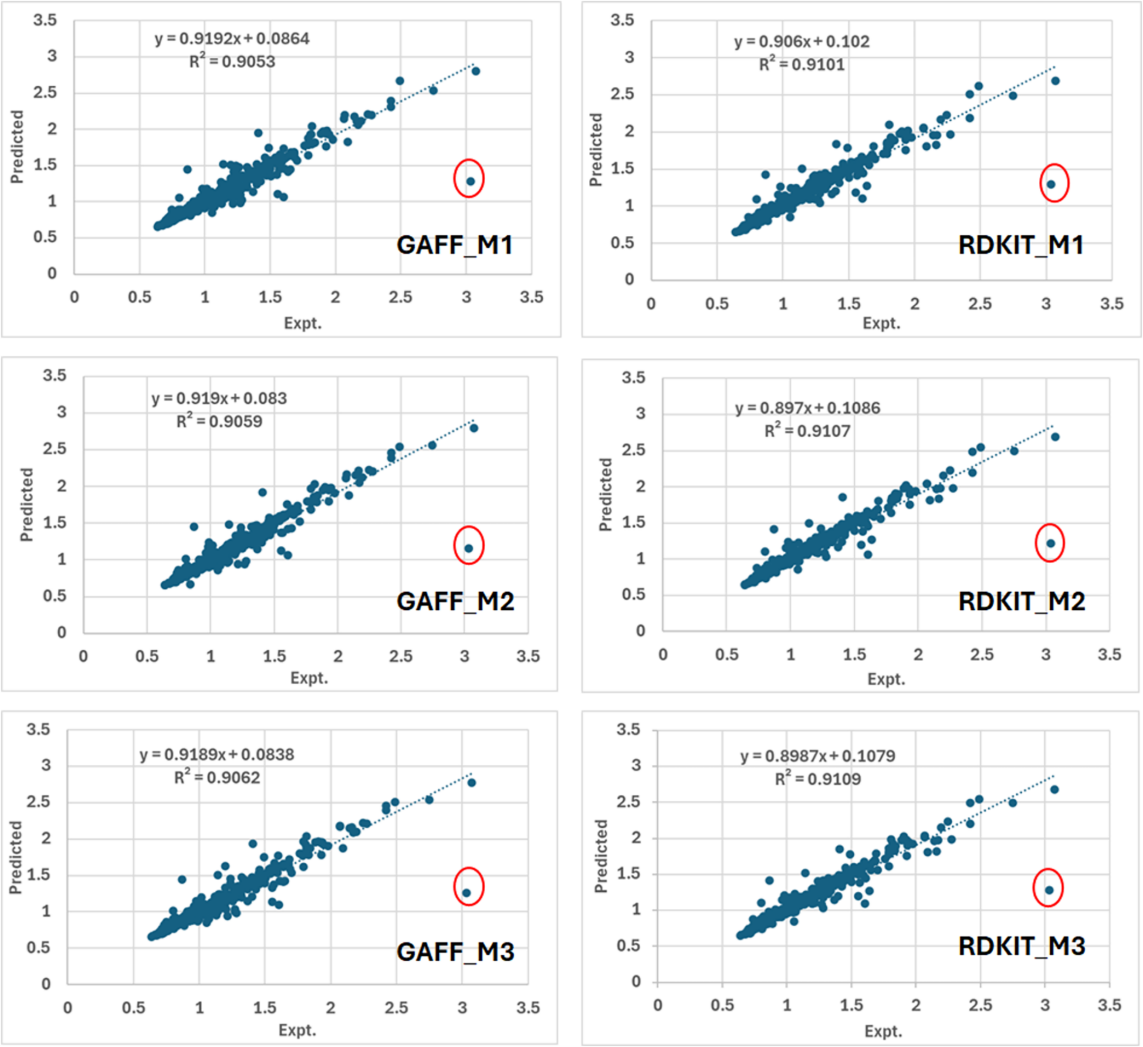
Scatter plots comparing predicted and experimental values for the test set, Set B using various machine learning models. For the M2 and M3 models, seven compounds were excluded due to missing temperature information. The compound highlighted with red circles is tetrafluoromethane (CAS 75-73-0). Although the CRC Handbook lists an experimental density of 3.034 at 25 °C, this compound exists as a gas at 25 °C and 1 atm.

**Table 4:** The Performance of Machine Learning Models for Density Prediction on Set C and Set D^a^ ^,^ ^b^

data set	model	no. data	ASE	APSE	AUE	RMSE	APE	*R* ^2^
Set C	GAFF_M1	188	–0.002	0.221	0.028	0.056	3.119	0.9618
Set C	GAFF_M2	188	–0.003	0.156	0.025	0.051	2.794	0.9680
Set C	GAFF_M3	188	–0.004	0.066	0.028	0.055	3.098	0.9639
Set C	RDKIT_M1	188	–0.004	0.098	0.024	0.048	2.653	0.9716
Set C	RDKIT_M2	188	–0.004	0.096	0.022	0.047	2.465	0.9732
Set C	RDKIT_M3	188	–0.002	0.217	0.023	0.047	2.546	0.9720
Set D	GAFF_M1	30	–0.111	–14.579	0.112	0.157	21.113	0.8894
Set D	GAFF_M2	30	–0.088	–11.014	0.091	0.136	17.866	0.8992
Set D	GAFF_M3	30	–0.096	–11.773	0.098	0.143	18.537	0.9004
Set D	RDKIT_M1	30	–0.120	–14.954	0.120	0.178	21.406	0.8630
Set D	RDKIT_M2	30	–0.098	–12.151	0.098	0.151	18.602	0.8865
Set D	RDKIT_M3	30	–0.110	–13.717	0.110	0.166	20.168	0.8765

a Definitions of performance metrics, ASE, APSE, AUE, RMSE, APE and R 2 are provided in the footnote.

b ASE, average difference; APSE, average percent difference; AUE, average unsigned error; RMSE, root mean square error; APE, average percent error; *R* ^2^, correlation coefficient square.

The ML models were applied to predict densities for two additional data sets: Set C, a subset of molecules used in molecular mechanics force field evaluation, and Set D, comprising molecules whose densities were measured at elevated pressures (>1 atm). As shown in Table 4, both models predicted Set C densities with reasonable accuracy. Specifically, GAFF_M1 achieved an RMSE of 0.056 g/cm^3^ and an APE of 3.119%, while RDKIT_M1 performed slightly better with an RMSE of 0.047 g/cm^3^ and an APE of 2.653%. In contrast, Set D densities were systematically overestimated by approximately 0.1 g/cm^3^, with APE values around 20%. This discrepancy is expected as the training data consisted exclusively of measurements at standard atmospheric pressure.

We next performed Shapley analysis for GAFF_M1 and RDKIT_M1. The key descriptors contributing to model performance, as indicated by Shapley values, are shown in Figure 2. Definitions of the RDKit descriptors (Figure 2, right panel) can be found on the RDKit Web site: br—bromine; ca.—aromatic carbon; c3—sp^3^ carbon; cl—chlorine; i—iodine; o—carbonyl oxygen; h1—aliphatic hydrogen attached to a sp^3^ carbon with one electron-withdrawing substituent; ha—aliphatic hydrogen attached to a carbon. NATOM represents the total number of atoms in the molecule.

**Figure 2:**
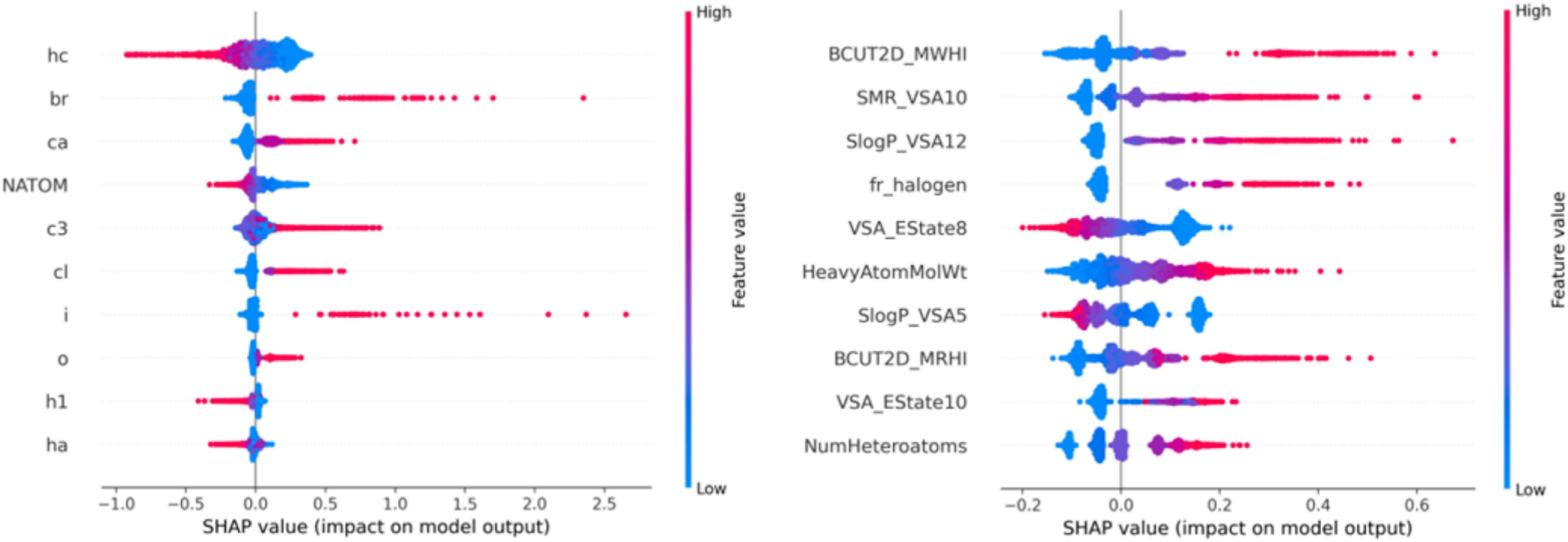
Summary of Shapley analysis for the density models GAFF_M1 (left) and RDKIT_M1 (right), illustrating how each descriptor contributes to and influences the predicted values.

We then performed a global sensitivity analysis (GSA) on the best-performing ML algorithms using both GAFF and RDKit descriptors. Because RDKit descriptors are highly correlated, the Shapley analysis is less effective at identifying the truly influential descriptors in these models. The GSA results are summarized in Figure 3 for GAFF and Figure S4 for RDKit. One additional atom type, ss, stands for sp^3^ sulfur in sulfide was identified by GSA. The baseline RMSE values used to identify key descriptors in the GSA were 0.0827 and 0.0791 g/mL for GAFF_M1 and RDKIT_M1, respectively. These values correspond to the cross-validation RMSEs obtained during model reoptimization.

**Figure 3:**
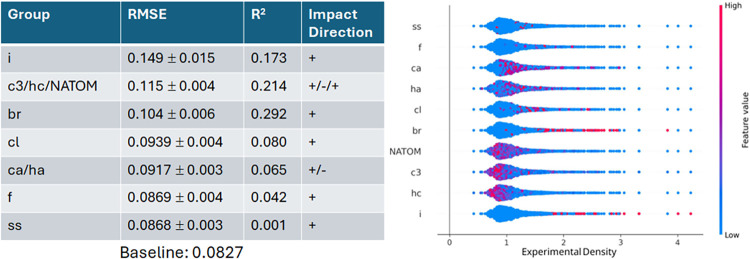
Summary of the global sensitivity analysis (GSA) for the density model GAFF_M1. The table on the left lists the impacts of key descriptors on RMSE and *R* ^2^, along with the direction of effect (“+” indicates that higher descriptor values increase the predicted value, whereas “–” indicates the opposite). The right panel shows the distributions of the top descriptors alongside the experimental values.

Both Shapley analysis and global sensitivity analysis (GSA) provide insight into the key contributors to model predictions. The observed trend that increased halogen content leads to higher density is physically reasonable. Descriptors corresponding to sp^3^-hybridized carbon (c3) and sp^2^-hybridized carbon (c2) contribute positively to density (positive SHAP values and “+” impact direction in GSA), as they generally increase molecular mass more than molecular volume in many organic systems. In contrast, hydrogen atoms bonded to c3 (hc) and c2 (ha) show consistently negative contributions (negative SHAP values and “–” impact direction in GSA), reflecting their minimal mass relative to the steric volume they occupy, which reduces the overall mass-to-volume ratio. In comparison, Shapley analysis and GSA based on RDKit descriptors are more difficult to interpret in terms of clear physical meaning, as these descriptors are often more abstract and less directly connected to fundamental molecular properties.

### 3.2 Refractive Index (nD) Prediction

We had a similar observation about the machine-learning algorithms for the refractive index (nD) property. The nD distribution of all of the compounds involved is exhibited in Figure S5. The separated data set for model training and validation (set E) conveys a similar data distribution to the test set (set F) regarding compound nD values, which is proper for the next step study. As listed in Table S6, the GPR algorithm family ranked top 1 followed by SVM, ensemble, and NN families for both the GAFF and RDKit descriptors. We selected rational quadratic GPR for GAFF and exponential GPR for RDKit to conduct 20-run model optimization with an aim to minimize the cross-validation RMSE. To demonstrate the robustness of our model training process for nD, we illustrated the distributions of the RMSE values of 20 independent training runs for M1 models and M2 and M3-series models introduced later (Figure S6). The corresponding mean and standard deviation values were displayed at the top of each chart. The best-performing models, named as GAFF_M1 and RDKIT_M1, were applied to make predictions for the test set, Set F. All the compound information (CAS ID, experimental nD, temperature of measurement, and Smiles) and the predicted nD for the training set (Set E) and test set (Set F) are listed in Tables S7 and S8, respectively.

The performances of GAFF_M1 and RDKIT_M1 are summarized in Table 5. RDKIT_M1 achieves slightly better performance than GAFF_M1, likely due to its use of a larger descriptor set (208 vs 47). Scatter plots comparing the experimental and predicted values are shown in Figure 4. Notably, the highlighted data points correspond to 1,2-diiodoethane (CAS 624-73-7), which exhibits significantly larger nD values than the rest of the data set.

**Figure 4:**
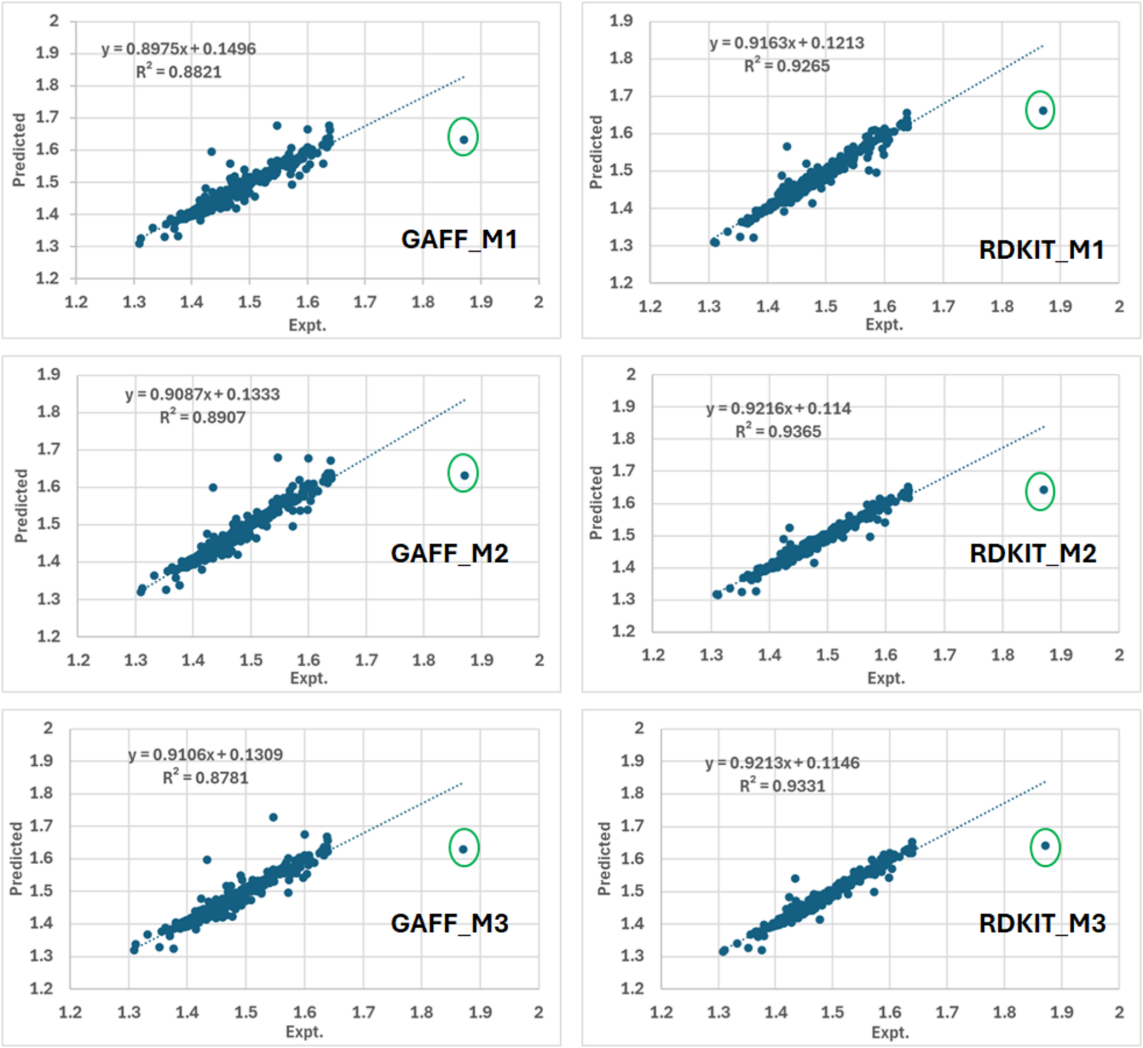
Scatter plots comparing predicted and experimental values for the test set, Set F across various machine learning models. Several compounds were excluded due to missing temperature information or failure to generate descriptors: two for RDKIT_M1 and 11 for all M2 and M3 models. The compound highlighted with green circles is 1,2-diiodoethane (CAS 624-73-7), which has a listed nD value of 1.871 at 20 °C, significantly larger than the rest of the compounds.

**Table 5:** Performance of Machine Learning Models for Refractive Index (nD) Prediction on Sets E and F^a^ ^,^ ^b^

data set	including NT data	model	no. data	ASE	APSE	AUE	RMSE	APE	*R* ^2^
Set E	Yes	GAFF_M1	3691	0.000	–0.015	0.008	0.016	0.558	0.9422
Set E	Yes	GAFF_M2	3628	0.000	–0.013	0.008	0.016	0.556	0.9456
Set E	Yes	GAFF_M3	3628	0.000	–0.016	0.009	0.017	0.613	0.9375
Set E	Yes	RDKIT_M1	3682	0.000	–0.008	0.004	0.011	0.301	0.9743
Set E	Yes	RDKIT_M2	3619	0.000	–0.010	0.006	0.013	0.399	0.9619
Set E	Yes	RDKIT_M3	3619	0.000	–0.011	0.006	0.014	0.426	0.9588
Set F	Yes	GAFF_M1	400	0.001	0.066	0.012	0.023	0.814	0.8821
Set F	Yes	GAFF_M2	391	0.001	0.052	0.011	0.022	0.767	0.8893
Set F	Yes	GAFF_M3	391	0.001	0.028	0.012	0.024	0.817	0.8762
Set F	Yes	RDKIT_M1	398	0.002	0.106	0.009	0.018	0.583	0.9265
Set F	Yes	RDKIT_M2	389	0.001	0.065	0.008	0.017	0.530	0.9365
Set F	Yes	RDKIT_M3	389	0.001	0.060	0.008	0.017	0.543	0.9331
Set E	No	GAFF_M1	3628	0.000	–0.008	0.008	0.016	0.544	0.9462
Set E	No	GAFF_M2	3628	0.000	–0.013	0.008	0.016	0.556	0.9456
Set E	No	GAFF_M3	3628	0.000	–0.016	0.009	0.017	0.613	0.9375
Set E	No	RDKIT_M1	3619	0.000	–0.005	0.004	0.011	0.297	0.9743
Set E	No	RDKIT_M2	3619	0.000	–0.010	0.006	0.013	0.399	0.9619
Set E	No	RDKIT_M3	3619	0.000	–0.011	0.006	0.014	0.426	0.9588
Set F	No	GAFF_M1	391	0.001	0.078	0.012	0.023	0.792	0.8858
Set F	No	GAFF_M2	391	0.001	0.052	0.011	0.022	0.767	0.8893
Set F	No	GAFF_M3	391	0.001	0.028	0.012	0.024	0.817	0.8762
Set F	No	RDKIT_M1	389	0.002	0.102	0.008	0.018	0.563	0.9282
Set F	No	RDKIT_M2	389	0.001	0.065	0.008	0.017	0.530	0.9365
Set F	No	RDKIT_M3	389	0.001	0.060	0.008	0.017	0.543	0.9331

a Definitions of performance metrics (ASE, APSE, AUE, RMSE, APE, and *R* ^2^) are provided in the footnote. NT denotes measurements without associated temperature data. The reported dataset sizes here correspond to the subset of molecules retained after descriptor generation; compounds for which descriptors could not be computed successfully were excluded from subsequent modeling.

b ASE, average difference; APSE, average percent difference; AUE, average unsigned error; RMSE, root mean square error; APE, average percent error; *R* ^2^, correlation coefficient square.

We conducted a Shapley analysis and GSA to identify the most influential descriptors for this property. Shapley analysis was performed on the best-performing model, while GSA was carried out for the best-performing ML algorithm. Figure 5 summarizes the Shapley analysis results for GAFF_M1 (left) and RDKIT_M1 (right). Atom type definitions follow those in Figure 2, with os representing sp^3^ oxygen in ethers and esters and c2 representing sp^2^ carbon in alkenes. GSA was conducted for both GAFF_M1 and RDKIT_M1, with the results shown in Figure 6. One additional atom type, cc (sp^2^ carbon in nonaromatic conjugated systems), was identified as a significant descriptor. It is reasonable to group c3/hc/NATOM and ca/ha together as c3/hc and ca/ha frequently appear within the same structural motifs, and the total count of c3/hc atoms correlates with the total number of atoms (NATOM) in a molecule.

**Figure 5:**
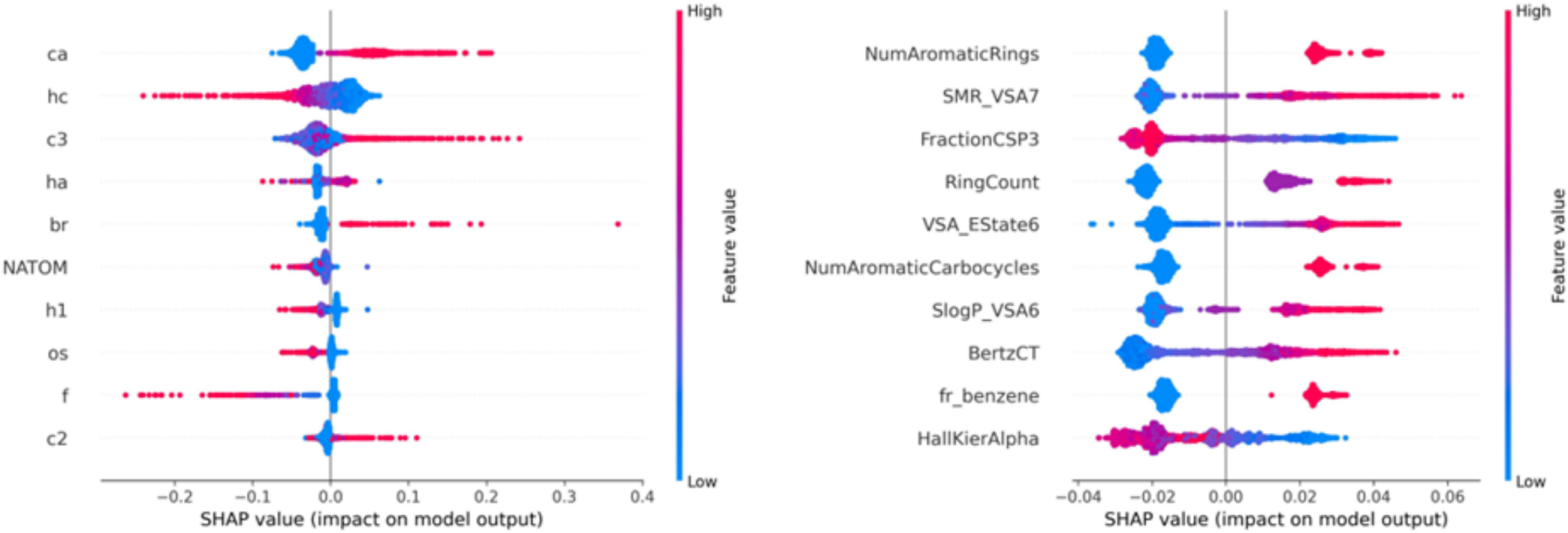
Summary of Shapley analysis for the nD models GAFF_M1 (left) and RDKIT_M1 (right), illustrating how each descriptor contributes to and influences the predicted values.

**Figure 6:**
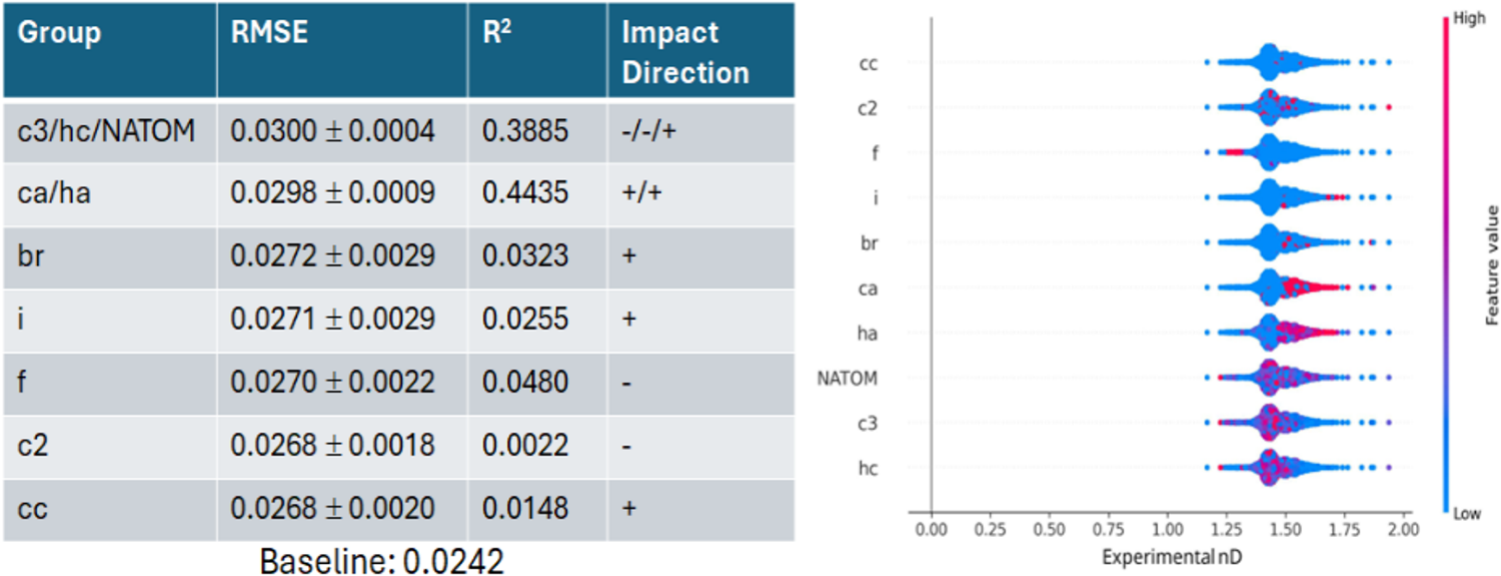
Summary of the global sensitivity analysis (GSA) for the nD model GAFF_M1. The table on the left lists the impacts of key descriptors on RMSE and *R* ^2^, along with the direction of effect (“+” indicates that higher descriptor values increase the predicted value, whereas “–” indicates the opposite). The right panel shows the distributions of the top descriptors alongside the experimental values.

Unlike density, the value of nD is governed primarily by electronic polarizability rather than mass. These trends can be rationalized by differences in polarizability: localized σ-bonded groups, such as sp^3^ carbons (c3) and their bonded hydrogens (hc), as well as weakly polarizable atoms such as fluorine, tend to decrease nD (“–” impact direction in GSA). In contrast, delocalized π-systems, including conjugated (cc) and aromatic carbons (ca), along with highly polarizable atoms such as bromine and iodine, contribute to increased nD (“+” impact direction in GSA). In the Shapley analysis, features with positive SHAP values (e.g., ca, c3, br, and i) contribute to increases in nD, whereas those with negative SHAP values (e.g., hc and f) contribute to decreases in nD. For sp^3^ carbons (c3) and their bonded hydrogens (hc), the net effect is typically a decrease in nD, as hc atoms generally outnumber c3 atoms in most molecules.

## 4 Discussion

### 4.1 Comparison with Public-Domain Models

This is the first paper in this series to enable on-the-fly prediction of solubility for arbitrary solutes in arbitrary solvents. The top-performing models (GAFF_M1 and GAFF_M2) show strong correlations between predicted and experimental values for both density and refractive index. The relatively large data sets used in this study (∼5000 compounds for density and ∼ 4000 for refractive index) enhance model robustness, and the low prediction errors further support their reliability. Using external test sets, our ML models achieved prediction errors of approximately 3% for density and 0.5% for refractive index, outperforming most previously published models summarized in Table 1.

As a representative comparison, the elemental-ratio-based model proposed by Kuwata et al.^13^ achieves an accuracy of 12% for compounds composed of C, H, and O. We applied the reported equation (Table S9) to 2,374 such compounds in our data set. The baseline model yields an accuracy of 13.28%, which improves to 12.27% after excluding 238 compounds with measured densities below 0.75 or above 1.9. For the model reported by Saldaña et al.,^15^ the predicted RMSE is 0.092 g/mL for 730 compounds. Overall, all machine learning-based models developed in this study outperform the baseline model and achieve performance comparable to that of the model by Saldana et al.,^15^ despite being trained and evaluated on a substantially larger data set.

Accurate prediction of neat liquid densities is also a central objective in the development of molecular mechanics force fields (MMFFs). In contrast to machine-learning approaches, MMFF-based methods are not inherently constrained by the scope of training data and, in principle, can provide more reliable predictions across a wide range of thermodynamic conditions, as demonstrated below. Using GAFF as an example, when combined with RESP charges, the predicted RMSE and APE are 0.036 g/mL and 2.21%, respectively, for 1,839 organic solvents.^34^ When ABCG2 charges are employed, the performance remains essentially unchanged with an RMSE of 0.037 g/mL and an APE of 2.24%.

### 4.2 Outliers and Further Data Curation

In comparison to experimental values, we identified a set of 72 outliers, which have the largest deviations. An outlier was recognized when its absolute percent error exceeded 9%. For example, tetrafluoromethane (CF_4_) was identified as an outlier. CF_4_ is a gas under ambient conditions and only liquefies at −183 °C with a density of 1.89 g/cm^3^, according to a material safety data sheet of chemicalbook (https://www.chemicalbook.com). Indeed, we conducted MD simulations at −183 °C and found that the predicted density of CF_4_ is 1.8571. Although an exact density value at 1 atm and room temperature is not available in the public domain, the listed experimental value of 3.034 g/cm^3^ is clearly inconsistent with the fact that CF_4_ is a gas. The density for CCl_4_ (1.59 g/cm^3^)^41^ and CBr_4_ (2.96 g/cm^3^)^42^ can further support that the experimental value of 3.034 is problematic.

We performed MD simulations to estimate the densities of the pure liquids for the outlier compounds. The ML-predicted values were generally closer to the MD results than to the originally reported experimental values. Motivated by this observation, we carried out further data curation and determined that most outliers were associated with questionable experimental records. Updated experimental values for 72 outlier compounds were identified through an extensive literature search. For some compounds, the temperatures of the new measurements differ from those in the original records. Table 6 summarizes the APE values among the original experimental data, revised experimental data, MD predictions, and ML predictions. Detailed information, including CAS numbers, experimental densities and measurement temperatures, SMILES strings, ML-predicted densities from all models, and sources for the revised experimental data, is provided in Table S10.

**Table 6:** Average Percent Errors (%) of ML-Predicted Density against Experimental Measurement and MD-Predicted Values for 72 Outliers

	expt	new expt	MD
expt		17.11	18.91
new expt	17.11		5.44
MD	18.91	5.44	
GAFF_M1	19.78	9.54	8.65
GAFF_M2	21.11	8.02	6.01
GAFF_M3	21.40	7.80	6.78
RDKIT_M1	5.77	17.64	19.23
RDKIT_M2	21.25	7.87	5.93
RDKIT_M3	21.25	7.67	6.44

It is noted that although alternative experimental density values for the outliers were identified through web searches, the original sources are largely unavailable. Therefore, these values should be interpreted with caution.

### 4.3 Role of Temperatures

For density, we compiled refined subsets of Sets A and B after excluding outliers and records lacking temperature information. The temperatures for experimental density measurement range from −104 to 242 °C, with an average of 23.9 °C. Two groups of models were then constructed: GAFF_M2 and RDKIT_M2 incorporate the experimental measurement temperature as an additional descriptor, whereas GAFF_M3 and RDKIT_M3 do not include temperature as an input descriptor. Similarly, for refractive index, we compiled refined subsets of Sets E and F by excluding records without temperature information. The temperatures for experimental nD measurement range from −100 to 134 °C, with an average of 22.7 °C. Two corresponding groups of models were then built: GAFF_M2 and RDKIT_M2 include temperature as an additional descriptor, while GAFF_M3 and RDKIT_M3 exclude temperature from the descriptor set. The performance of the M2 and M3 models is summarized in Tables 3–5. It is evident that including temperature information improves model performance, as seen when comparing the corresponding M2 and M3 models across both descriptor types and molecular properties. Additionally, the M3 models generally outperform the corresponding M1 models, particularly for density. This demonstrates that ML models can achieve higher accuracy after removing outliers with questionable experimental values. For density prediction using GAFF descriptors, the M2 and M3 models achieve APEs of 3.07 and 3.21, respectively, for Set B, and 2.79 and 3.10 for Set C. Models built with RDKit descriptors show better predictive performance, with M2 and M3 APEs of 2.67 and 2.86 for Set B and 2.46 and 2.55 for Set C. For refractive index (nD) prediction, GAFF-based models yield APEs of 0.77 and 0.82, whereas RDKit-based models achieve lower APEs of 0.53 and 0.54 for M2 and M3, respectively.

However, for the M1 and M3 models, density cannot be predicted at different temperatures because temperature is not included as a descriptor. Moreover, for the M2 models, reliable predictions are not expected at temperatures far from ambient conditions, as most measurements were conducted near room temperature (23.9 ± 17.0 °C). This limitation reflects a fundamental constraint of machine learning approaches: without sufficient temperature-resolved data for individual compounds, it is difficult to develop reliable models for temperature-dependent density prediction. In contrast, physics-based methods, such as molecular dynamics simulations using classical force fields, can more robustly predict densities over a wide temperature range. A similar limitation applies to refractive index prediction.

### 4.4 Feature Analysis

Compared with the feature-importance algorithms available in MATLAB (e.g., F-test and MRMR), which are primarily designed for linear regression models, the methods used in this study, Shapley analysis and global sensitivity analysis (GSA), are applicable to a wide range of machine learning models and are not restricted to linear ones. The kernel SHAP algorithm adopted here employs a conditional value function, explicitly accounts for feature interactions, and provides a more accurate assessment of the descriptor importance. However, it is inefficient in handling highly correlated descriptors, and its main limitation is its substantially higher computational cost. In contrast, GSA more effectively reveals the collaborative relationships among descriptors by quantifying the influence of each descriptor group on the overall model performance, thereby highlighting the indispensability of a descriptor group relative to others. Another key difference is that Shapley analysis identifies important contributors at the level of individual data entries, and statistical analysis of Shapley values is then used to infer the globally important descriptors. GSA, on the other hand, identifies key contributors directly at the level of feature groups by using all data entries simultaneously.

As a result, Shapley analysis is more suitable for problems in which the number of descriptors (*m*) is much larger than the number of data entries (*n*), whereas GSA is better suited for cases with *n* ≫ *m*. The density and refractive index (nD) data sets used in this work fall into the latter category.

Examination of the major contributors identified by Shapley analysis and GSA indicates that density is primarily governed by mass packing and spatial efficiency, whereas refractive index is largely determined by electronic polarizability and π-conjugation effects. These distinctions account for the differing trends observed for sp^3^ and sp^2^ carbons and their bonded hydrogens.

### 4.5 Machine Learning Algorithm Family

We evaluated the performance of each machine-learning algorithm family in building predictive models using 10-fold cross-validation RMSE as the metric. For density prediction with GAFF descriptors, the Gaussian process regression (GPR) family ranked first in terms of RMSE, followed by neural networks (NNs). The third-best family was ensemble for model M1 and SVM for models M2 and M3. For RDKit descriptors, GPR again ranked first across all of the models. For M1, ensemble and NN ranked second and third, respectively; for M2, NN and SVM ranked second and third; and for M3, ensemble and SVM ranked second and third.

A clearer pattern was observed for the refractive index (nD) prediction. With GAFF descriptors, the top three algorithm families were consistently GPR, NN, and SVM for models M1, M2, and M3. With RDKit descriptors, GPR and SVM ranked first and second for all of the three models. The third-ranked family was ensemble for M1 and NN for both M2 and M3.

### 4.6 Descriptors

In this study, both GAFF and RDKit descriptors were used to construct ML models. GAFF descriptors are purely atom-based and capture subtle local chemical environments within a molecule, whereas RDKit descriptors include not only atom-based features but also global molecular properties. GAFF provides 47 descriptors, which is substantially fewer than the 208 descriptors available in RDKit. Overall, RDKit-based models show better predictive performance than the corresponding GAFF-based models for both the density and refractive index (nD).

However, RDKit-based models may be less robust than GAFF-based models. For density prediction, many outliers identified by the GAFF_M1 model in Set A were associated with inconsistent experimental values, whereas the RDKit_M1 model detected only a few outliers in the same set. In other words, RDKit_M1 produced strong predictions even when trained on data sets containing unreliable or inconsistent experimental measurements. Another limitation of RDKit descriptors is that descriptor generation fails for more compounds compared with GAFF, for which atom types were assigned using the Antechamber module^39^ in AMBER.

### 4.7 Future Directions in Organic Process Development

Data-driven prediction of density and refractive index provides an efficient means to accelerate organic process development workflows. Reliable in silico estimates can inform solvent and solvent mixture selection, supply prior knowledge for process analytical technology (PAT) calibration, and support early-stage process design decisions when experimental characterization is limited or unavailable. In this context, predictive models that generalize across diverse organic chemistries and require minimal user input are particularly valuable for process chemists and engineers. By enabling rapid, on-demand access to these process-relevant physical properties, the models described in this work complement experimental measurements and offer a practical tool for supporting process development, scale-up, and manufacturing decision-making. Extension of the present framework to multicomponent systems represents a natural next step, and the machine learning models developed here provide a solid foundation for predicting the density and refractive index of organic mixtures.

## 5 Conclusion

We explored a broad pool of machine-learning (ML) algorithms for predicting density and refractive index (nD) using expanded data sets. Among all algorithm families tested, Gaussian process regression (GPR) consistently delivered the best performance based on 10-fold cross-validation root-mean-square error (RMSE). The resulting ML models achieved density prediction accuracy with an average percent error (APE) of about 3%, comparable to that obtained from physics-based approaches such as molecular dynamics simulations with molecular mechanics force fields.^34^ For nD prediction, the APE was substantially lower, at approximately 0.6%. Temperature was identified as a key descriptor for density prediction. When temperature information is available, the RDKIT_M2 or GAFF_M2 models are recommended. When temperature is unavailable, RDKIT_M3 or GAFF_M3 should be used instead, as they were trained on curated data sets and demonstrate better performance than RDKIT_M1 and GAFF_M1. Feature analysis was performed using both Shapley analysis and global sensitivity analysis (GSA). GSA proved to be more computationally efficient and identified more statistically significant descriptors than Shapley analysis for the two molecular properties. Data curation was found to be critical for building accurate and reliable models. Our ML workflow identified 72 outliers, each of which has alternative experimental values reported in independent public sources. These updated values show much better agreement with both our ML predictions and molecular dynamics simulation results. The high accuracy of the ML models for both properties supports reliable prediction of molecular polarizability and other solvation free energy-related properties, representing a meaningful step toward on-the-fly prediction of solubility for arbitrary solute–solvent pairs. These models also lay the groundwork for developing protocols to predict density and refractive index for organic mixtures.

## Supplementary Material























## Data Availability

All experimental data came from the CRC Handbook of Chemistry and Physics [92nd ed. 2011–2012]. All of the prediction results were generated utilizing MATLAB 2024a software, and data processing was conducted using R 4.2.3. The data sets and best-performing models are available in a public GitHub repository (https://github.com/ClickFF/density-nD) in MATLAB session (.mat) format.

## Notes

Figure S1, which illustrates the importance of density and refractive index predictions in solvation free energy calculations; Figures S2 and S5, which present histogram distributions of experimental density and nD values, respectively; Figures S3 and S6, which show the distribution of RMSE values across 20 independent training runs for the best-performing machine learning models for density and nD, respectively; Figures S4 and S7, which show the distributions of the top descriptors in the RDKIT_M1 density and nD models alongside the corresponding experimental values (PDF)
Summarizing the performance of 28 machine-learning algorithms for density (Table S1) (XLSX)
It also provides compound information and predicted values from all six ML models (GAFF_M1, GAFF_M2, GAFF_M3, RDKIT_M1, RDKIT_M2, and RDKIT_M3) for density (Table S2 for Set A) (XLSX)
It also provides compound information and predicted values from all six ML models (GAFF_M1, GAFF_M2, GAFF_M3, RDKIT_M1, RDKIT_M2, and RDKIT_M3) for density (Table S3 for Set B) (XLSX)
It also provides compound information and predicted values from all six ML models (GAFF_M1, GAFF_M2, GAFF_M3, RDKIT_M1, RDKIT_M2, and RDKIT_M3) for density (Table S4 for Set C) (XLSX)
It also provides compound information and predicted values from all six ML models (GAFF_M1, GAFF_M2, GAFF_M3, RDKIT_M1, RDKIT_M2, and RDKIT_M3) for density (Table S5 for Set D) (XLSX)
Summarizing the performance of 28 machine-learning algorithms for density and refractive index, nD (Table S6) (XLSX)
It also provides compound information and predicted values from all six ML models (GAFF_M1, GAFF_M2, GAFF_M3, RDKIT_M1, RDKIT_M2, and RDKIT_M3) for density (Table S7 for Set E) (XLSX)
It also provides compound information and predicted values from all six ML models (GAFF_M1, GAFF_M2, GAFF_M3, RDKIT_M1, RDKIT_M2, and RDKIT_M3) for density (Table S8 for Set F) (XLSX)
Table S9 summarizes the densities of compounds containing C, H, and O predicted using the elemental ratio-based method (baseline model) (XLSX)
Table S10 provides compound information, model predictions, alternative experimental values, and corresponding literature sources for the 72 identified density outliers (XLSX)
