# Supplementary material for: Toward Predicting Solubility of Arbitrary Solutes in Arbitrary Solvents: Prediction of Density and Refractive Index Using Machine Learning Algorithms with Global Sensitivity Analysis
Source: Org Process Res Dev. 2026 Jun 12;30(7):2028–40. doi: 10.1021/acs.oprd.6c00166 (PMC13386533; doi:10.1021/acs.oprd.6c00166)
Supplement: Supplementary file 1 [file op6c00166_si_001.pdf]

Supporting Information

Toward Predicting Solubility of Arbitrary Solutes in Arbitrary Solvents: Prediction of Density and Refractive Index Using Machine Learning Algorithms with Global Sensitivity Analysis

Brian Hu[#a], Jingchen Zhai[#a], Xiguang Qi[a], Xibing He[a], Nick X. Wang,*[b] Junmei Wang[a]*

[a] *Department of Pharmaceutical Sciences and Computational Chemical Genomics Screening Center, School of Pharmacy, University of Pittsburgh, Pittsburgh, PA 15261, USA.*

[b] *Small Molecule Discovery, Eli Lilly and Company, Lilly Corporate Center, Indianapolis, Indiana 46285, USA.*

[#]: equal contributors

*: corresponding author, junmei.wang@pitt.edu, wang_nick@lilly.com

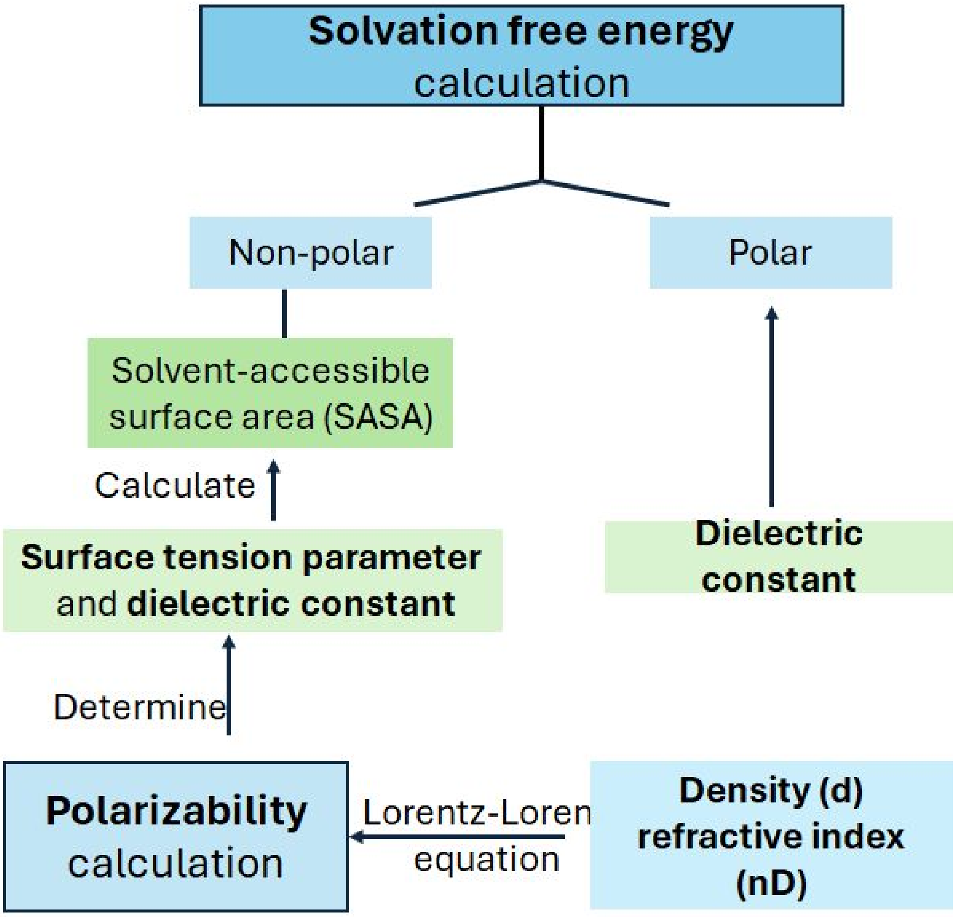

Figure S1. The significance of density and refractive index prediction in solvation free energy calculation for an arbitrary solute-solvent pair. models.

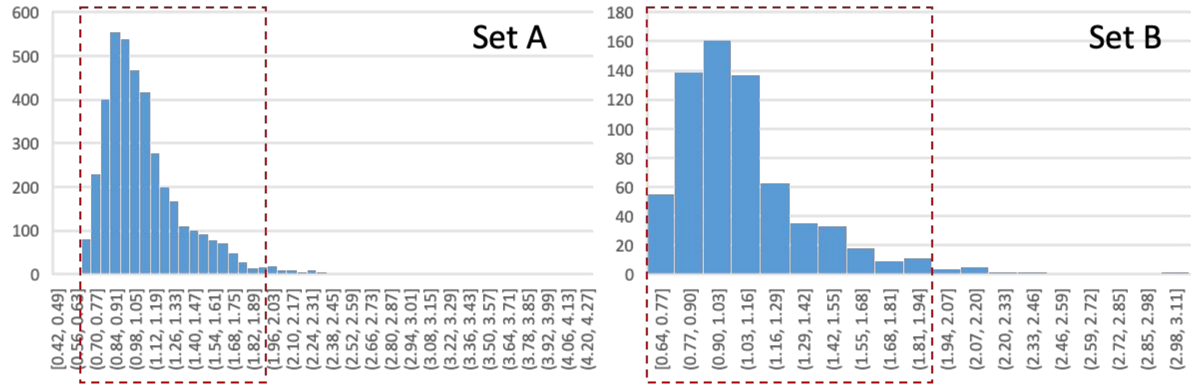

Figure S2. Histogram distributions of measured densities for compounds in Set A and Set B. The x-axis represents density values and the y-axis shows counts. In both the training set (Set A) and test set (Set B), most compounds fall within the range of 0.64–1.90 g/cm³.

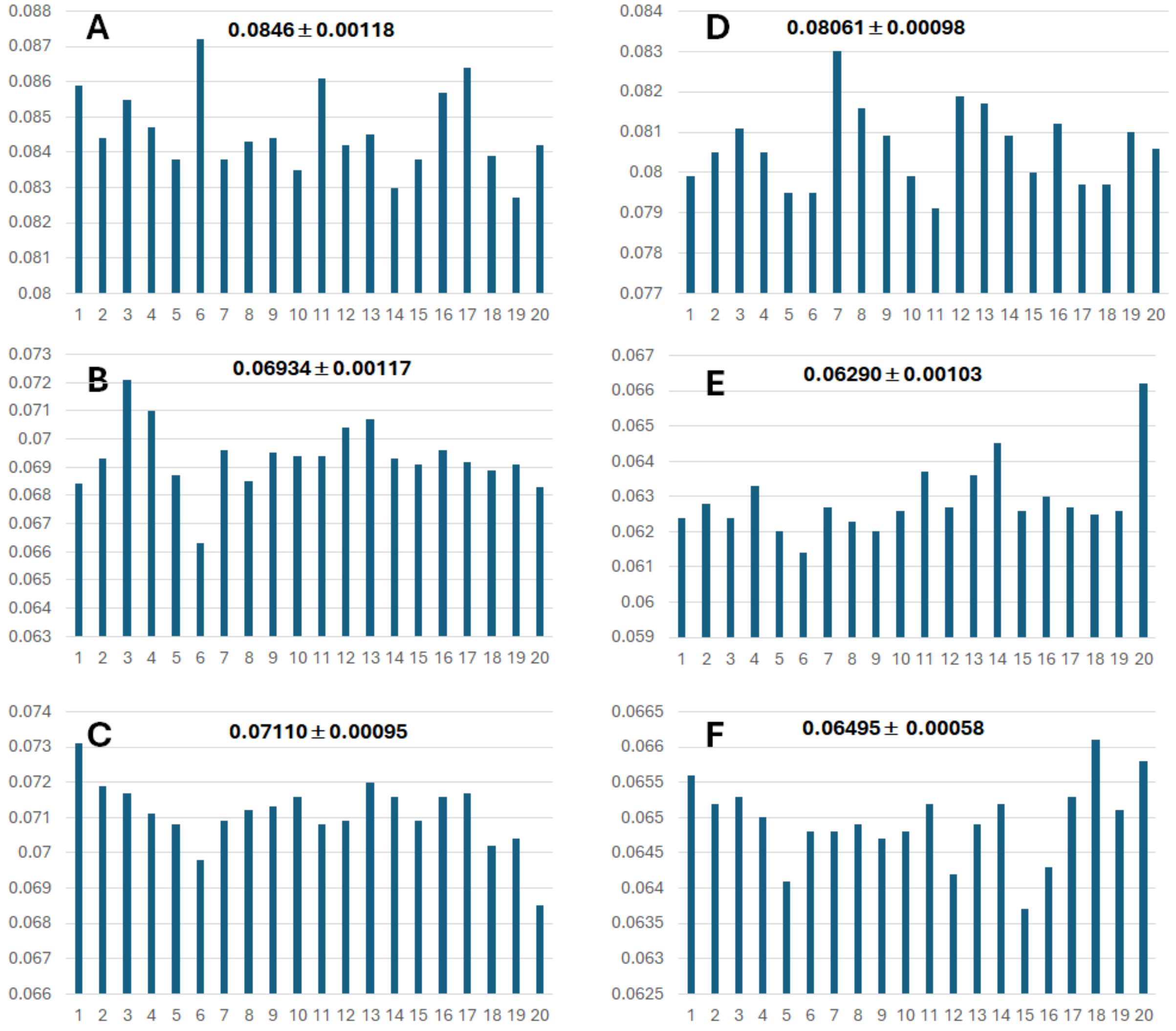

Figure S3. Distribution of RMSE values (Y-axis) across 20 independent training runs (X-axis) for the best-performing machine learning algorithm, Gaussian process regression (GPR), in density prediction. The mean and the standard deviation are shown on the top. (A) GAFF_M1, (B) GAFF_M2, (C) GAFF_M3, (D) RDKIT_M1, (E) RDKIT_M2, and (F) RDKIT_M3.

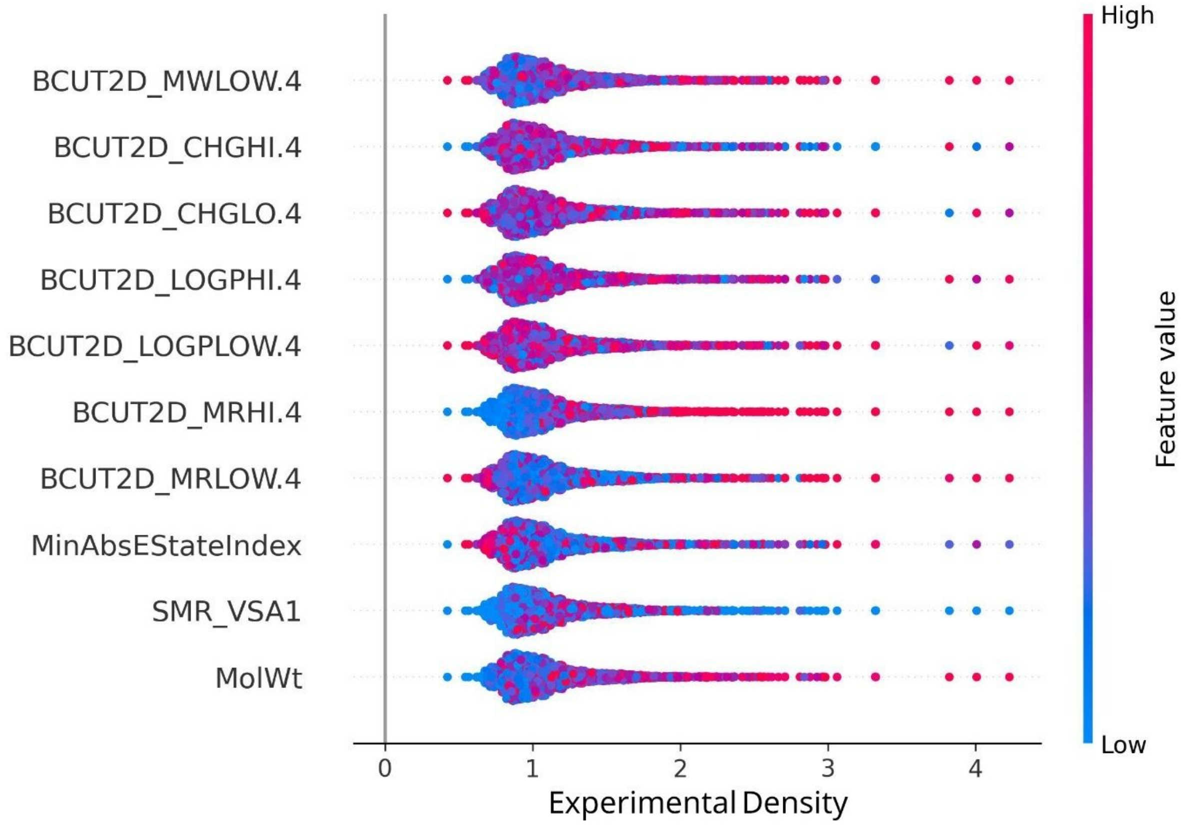

Figure S4. Distributions of the top descriptors in the RDKIT_M1 density model shown alongside the experimental values. The baseline cross-validation RMSE (0.0791 g/cm³) was used as the threshold for identifying key descriptors.

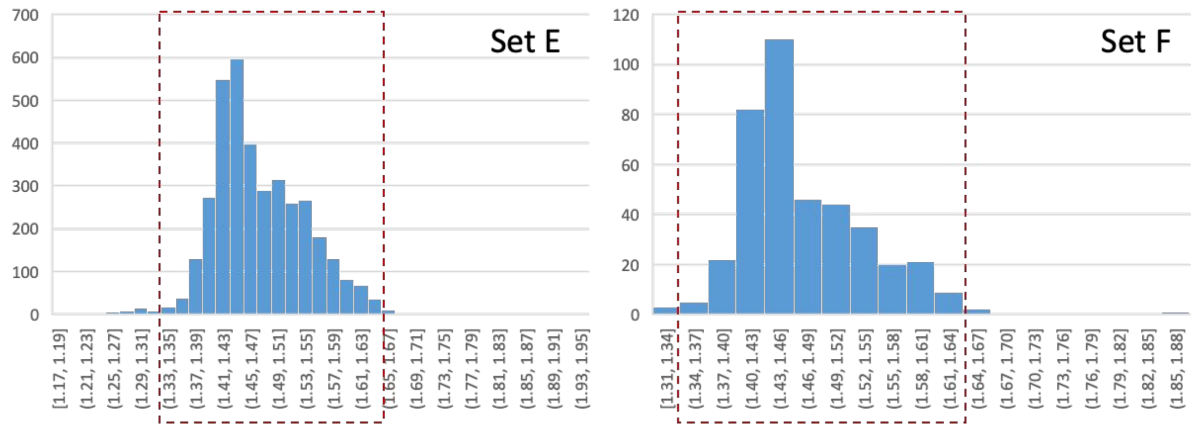

Figure S5. Histogram distributions of measured nD for compounds in Set A and Set B. The x-axis represents nD values and the y-axis shows counts. In both the training set (Set A) and test set (Set B), most compounds fall within the range of 1.34–1.64.

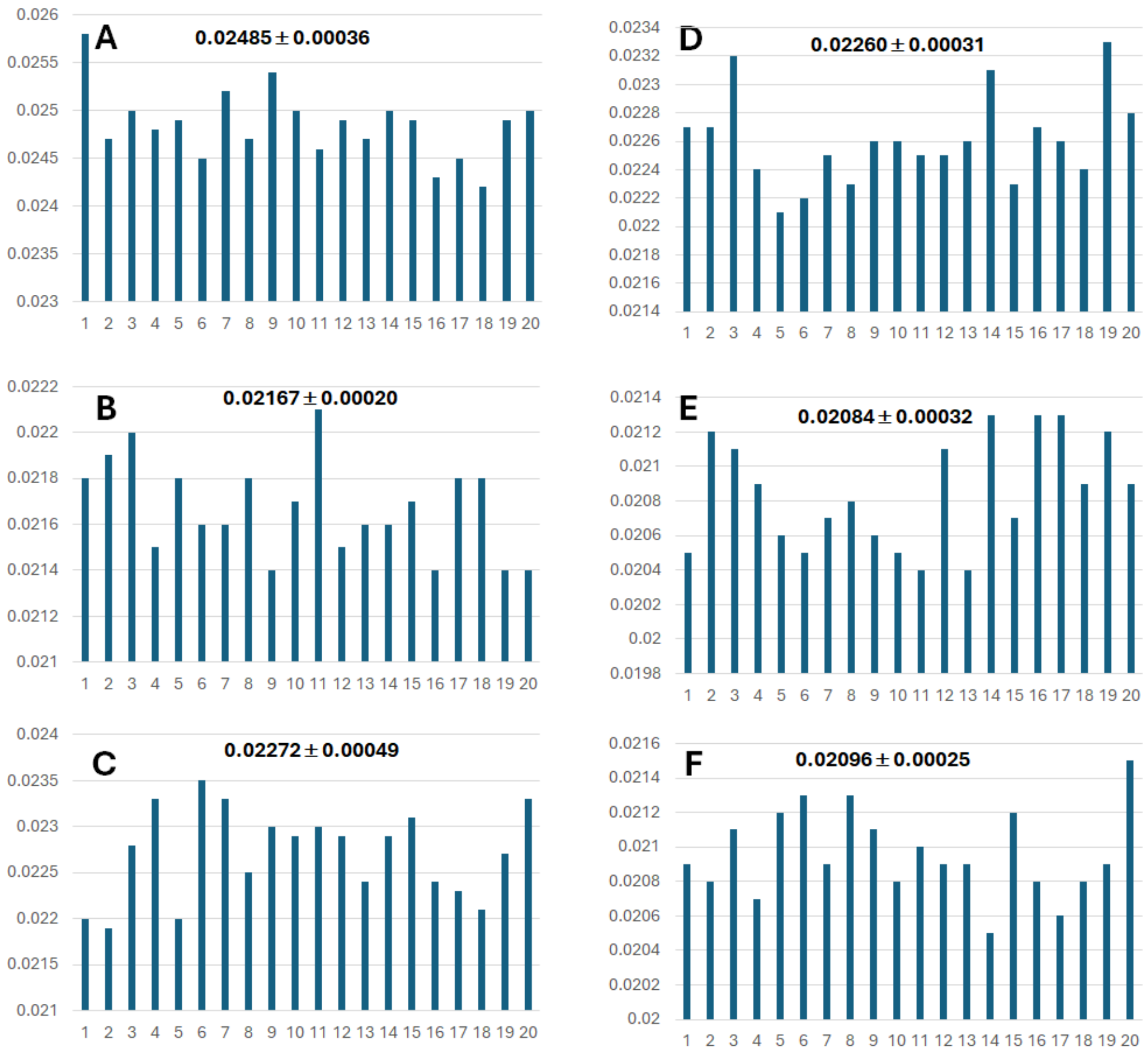

Figure S6. Distribution of RMSE values (Y-axis) across 20 independent training runs (X-axis) for the best-performing machine learning algorithm, Gaussian process regression (GPR), in nD prediction. The mean and the standard deviation are shown on the top. (A) GAFF_M1, (B) GAFF_M2, (C) GAFF_M3, (D) RDKIT_M1, (E) RDKIT_M2, and (F) RDKIT_M3.

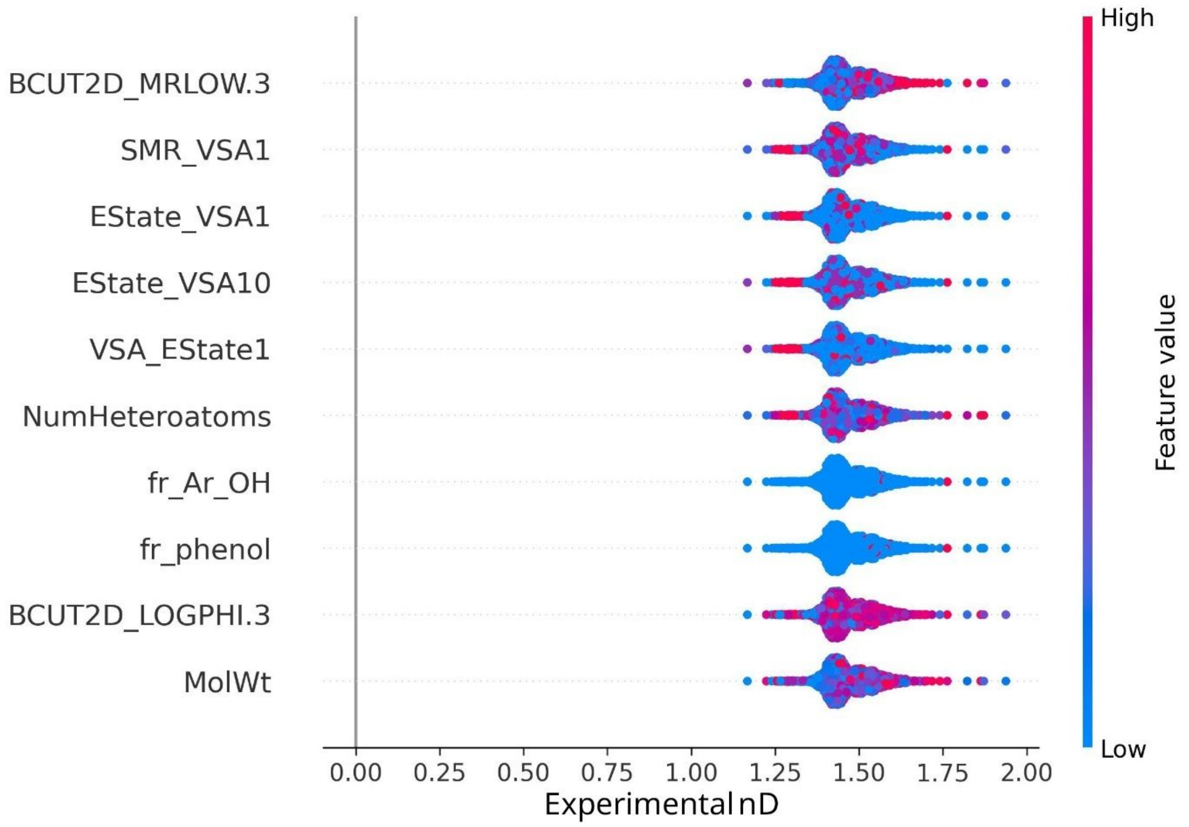

Figure S7. Distributions of the top descriptors in the RDKIT_M1 nD model shown alongside the experimental values. The baseline cross-validation RMSE (0.0221) was used as the threshold for identifying key descriptors.
